# The Borderline Bias in Explicit Emotion Interpretation

**DOI:** 10.3389/fpsyg.2021.733742

**Published:** 2021-12-15

**Authors:** Sylwia Hyniewska, Joanna Dąbrowska, Iwona Makowska, Kamila Jankowiak-Siuda, Krystyna Rymarczyk

**Affiliations:** ^1^Department of Experimental Psychology, University College London, London, United Kingdom; ^2^Psychiatric Clinic I, Institute of Psychiatry and Neurology, Warsaw, Poland; ^3^Child and Adolescent Psychiatric Department, Medical University of Łódź, Łódź, Poland; ^4^Department of Biological Psychology, Behavioral Neuroscience Lab, SWPS University of Social Sciences and Humanities, Warsaw, Poland

**Keywords:** borderline personality, emotion bias, face interpretation, nonverbal communication, emotion perception

## Abstract

Atypical emotion interpretation has been widely reported in individuals with borderline personality disorder (iBPD); however, empirical studies reported mixed results so far. We suggest that discrepancies in observations of emotion interpretation by iBPD can be explained by biases related to their fear of rejection and abandonment, i.e., the three moral emotions of anger, disgust, and contempt. In this study, we hypothesized that iBPD would show a higher tendency to correctly interpret these three displays of social rejection and attribute more negative valence. A total of 28 inpatient iBPDs and 28 healthy controls were asked to judge static and dynamic facial expressions in terms of emotions, valence, and self-reported arousal evoked by the observed faces. Our results partially confirmed our expectations. The iBPD correctly interpreted the three unambiguous moral emotions. Contempt, a complex emotion with a difficulty in recognizing facial expressions, was recognized better by iBPD than by healthy controls. All negative emotions were judged more negatively by iBPD than by controls, but no difference was observed in the neutral or positive emotion. Alexithymia and anxiety trait and state levels were controlled in all analyses.

## Introduction

Adaptive emotion interpretation is fundamental for healthy human interactions and the mental health of individuals. Atypical appraisal of emotional cues of others could be related to traits, such as anger, anxiety, and alexithymia (Schlegel et al., 2017; Kiliç et al., 2020), and are characteristics of various mental disorders, such as borderline personality disorder (BPD) (Domes et al., 2009, 2011; De Panfilis et al., 2015). Interestingly, research into emotion perception in individuals with borderline personality disorder (iBPD) reported heterogeneous results, with different studies suggesting deficits in emotion understanding, generalized negative biases, or, in some cases, even high sensitivity and more accurate labeling of subtle emotions.

Studies highlighting sensitivity to emotional signals, i.e., low threshold for the naming of emotional stimuli, showed, for example, that iBPD were able to correctly classify emotions at a lower intensity level of facial expression compared with healthy individuals (Lynch et al., 2006). Higher emotional reactivity in iBPD compared with controls was also reported as greater amygdala activation to emotional and neutral faces (Donegan et al., 2003), as well as to aversive stimuli in general (Herpertz et al., 2001). However, in several studies, neither increased psychophysiological responses, e.g., no greater potentiation of the startle response to negative pictures (Herpertz et al., 1999, 2000), nor any increased facial mimicry to facial expressions of emotions was observed (Matzke et al., 2014) in iBPD compared with controls. The authors of the latter study observed, however, a general tendency in iBPD to react with augmented activation of the corrugator supercilii muscle, i.e., frowning, to all displays of negative expressions. The authors concluded that, rather than heightened affective empathy in iBPD, a potential negativity bias could explain the diverse emotion interpretation deficits reported in the literature (Matzke et al., 2014). Moreover, visual search tasks where iBPD had to spot schematic happy and angry faces among neutral ones did not show any higher performance to angry stimuli compared with healthy participants either (Hagenhoff et al., 2013). Although only one negatively valenced emotion was presented, the authors suggested that these visual search results most probably can be explained by a lack of bias to negative stimuli.

Different studies also showed more general deficits in the interpretation of emotional displays. Deficits were observed in the naming of surprise when iBPD watched morphs from neutral to basic emotion displays and, after having reported the face to be emotional, were asked to attribute one of the six basic emotions (Domes et al., 2008). However, the authors did not provide any explanation of confusions or eventual biases observed in their study. Another study on morphs showed biases in the attribution of anger, with iBPD being more likely to respond “anger,” when anger and disgust faces were blended 50%/50% or anger and happiness faces were blended 40%/60% (Domes et al., 2008). Other teams reported deficits in the interpretation of negatively valenced emotional displays (Levine et al., 1997; Wagner and Linehan, 1999; Bland et al., 2004), which is sometimes interpreted as contradicting the existence of an increased vigilance to social threat stimuli as postulated by Linehan (1993). Some questions were raised, however, regarding the stimuli used, e.g., Bland et al. (2004) reported changes in anger, sadness, and disgust, but in each case, only one picture of the three presented for each emotion led to interpretation differences in iBPD compared with healthy participants, and the authors presented no confusion matrix. Another study showed a higher tendency to attribute fear to emotional displays and to neutral faces, which led to a higher correct attribution of fear in iBPD compared with healthy participants, as well as to a high number of false alarms when appraising neutral faces (Wagner and Linehan, 1999). Although only fear showed these results, the authors interpreted them as reflecting a negativity bias in iBPD when appraising social cues.

Different teams tried to explain the inconsistencies through the prism of comorbidities at play in BPD, particularly elevated alexithymia (Domes et al., 2011; Kiliç et al., 2020), or anxiety, which is reported at high levels in this population (Domes et al., 2008).

Another approach to elucidating emotion interpretation skills is to look at the characteristics of emotional stimuli encountered in naturalistic settings, i.e., before all the dynamic modality. Previous studies that have attempted to investigate the question of dynamic facial expression being easier to interpret have yielded inconsistent findings (for a review, see Kätsyri, 2006; Fiorentini and Viviani, 2011; Alves, 2013; Krumhuber et al., 2013; Rymarczyk et al., 2016). Interestingly, however, clinical and neuropsychological conditions have been shown to influence the extent to which dynamic displays lead to processing benefits (Ambadar et al., 2005; Torro-Alves et al., 2016; Bala et al., 2018; Żurowska et al., 2018). Thus, individuals with major depression have been observed to present atypical emotion interpretation patterns depending on whether they watched static or dynamic facial expressions of emotions, namely, greater accuracy in labeling static sadness and angry faces and less accuracy in labeling dynamic happiness faces (Bomfim et al., 2019). Patients with brain lesions in the mesial temporal zone have shown lower performance in interpreting social information from movement compared with healthy individuals (Bala et al., 2018). Patients during and after benzodiazepine detoxification recognized dynamic facial displays better than static displays, whereas no similar emotional recognition enhancement for the dynamic modality was observed in the healthy controls (Żurowska et al., 2018). Although numerous empirical scientists support the importance of testing both static and dynamic stimuli to improve the understanding of processes at play in emotion interpretation, whether in healthy or clinical populations, no study so far has investigated this aspect in the population with BPD.

Therefore, we decided to investigate how the presentation modality of stimuli, i.e., dynamic vs. static facial displays, affects emotion interpretation in BPD while controlling for alexithymia and anxiety levels. Given that fear of rejection and abandonment might be a defining feature of iBPD (Gunderson, 2008; De Panfilis et al., 2015), presenting iBPD with stimuli relating to these specific emotions would be invaluable for the understanding of atypical emotion perception in this disorder. In fact, most of the former studies in iBPD investigated some or all of the basic emotions as well as neutral faces and, to the best of our knowledge, have never included any expressions of contempt.

Although we do not have any expectations regarding how the presentation modality of stimuli might influence emotion interpretation in iBPD, we predict that facial expressions of emotions associated with social threat might exhibit a high unbiased hit rate, mostly anger, disgust, and contempt, the three being the so-called moral emotions (Hutcherson and Gross, 2011). Given the mentioned fear of rejection and abandonment, we expected higher arousal and higher negativity to be attributed to these three emotion stimuli by iBPD compared with healthy controls.

## Materials and Methods

### Participants

A total of 28 inpatients, meeting the diagnosis of BPD according to the *DSM-5* criteria, and 28 healthy individuals participated in this study. The sample size of this study was determined regarding an *a priori* power analysis, for which we used the G*Power software application (version 3.1.9.2, Heinrich-Heine-Universität Düsseldorf, Düsseldorf, Germany; Faul et al., 2007). According to the calculations, 17 samples per group were required to accomplish an ANOVA with an α of 0.05, a power (1 − β) of 0.80, and an effect size *f* of 0.40 based on the data provided in similar designs comparing emotion recognition in patients with BPD and healthy controls (Fenske et al., 2015; Lowyck et al., 2016; Kiliç et al., 2020). These seemed to be the only similar studies to report sufficient prior information to run power analyses. Participants from both groups were of similar age (*t* = − 1.77, *p* = 0.083, *d* = − 0.473).

All patients (24 females and 4 males), aged 19–57 years (M = 26.86; SD = 8.78, SE = 1.66), were referred to the study by psychiatrists from the 24/7 Department of Neurosis, Personality Disorders and Eating Disorders at the Institute of Psychiatry and Neurology in Warsaw, Poland. The higher ratio of females to males is a reflection of this specific patient population and in accordance with the *DSM-5*, which records a higher prevalence of women among those being diagnosed clinically with BPD. For the control group, individuals were involved from the general population in Warsaw (23 females and 5 males), aged 18–54 years (M = 30.96; SD = 8.60, SE = 1.62), through online advertisements and mailing groups. None from the control group had any current or past history of mental health conditions, nor any excessive consumption of alcohol or recreational drugs as verified through self-reports.

### Procedure

Videos and static photographs of forward-facing actors (two women and two men) were presented in a semi-random sequence. Each actor displayed nine emotional faces (i.e., joy, sadness, anger, fear, disgust, surprise, embarrassment, contempt, and pride) and one neutral face in dynamic and static format from the Amsterdam Dynamic Facial Expression Set (ADFES; Van der Schalk et al., 2011). In the neutral ADFES dynamic condition, actors could be observed blinking, closing their eyes, or slightly changing the position of their heads. All stimuli were 576 pixels in height and 720 pixels in width, presented on a gray background. For our study, facial displays from four ADFES actors were selected (two males and two females), presenting each emotion once in a dynamic format and once in a static format. Both formats are accessible in a usable form directly from the ADFES dataset. All stimuli were unambiguous, and as per the wish of authors, the expressions were to be highly standardized: they were to be included in the dataset exclusively when following closely established atheoretical prototypes for each emotion display and have received very high recognition rates in healthy populations (see Van der Schalk et al., 2011).

**FIGURE 1:**
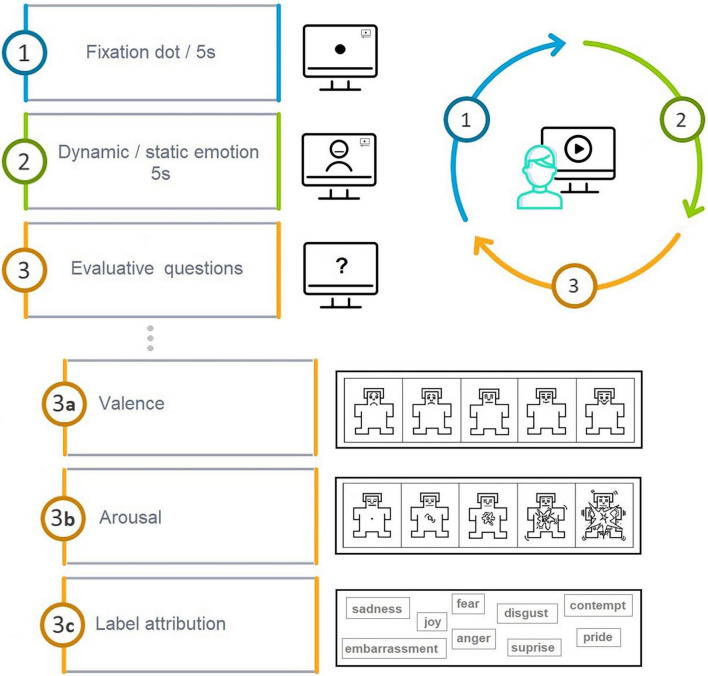
Experimental procedure.

Each participant saw and evaluated 80 stimuli in total (10 facial displays × 4 actors × 2 modalities). The experimental session was preceded by an explanatory session with two faces to be judged in order for the participants to become acquainted with the experimental procedure. Each face stimulus was preceded by a fixation dot (5 s duration) presented in the place where the face of the stimulus actor would follow (Figure 1). Each stimulus was presented for 5 s independently of whether in a photograph or video format. Each facial stimulus was followed automatically by three evaluative questions.

First, participants were asked to use the pictorial Self-Assessment Manikin (SAM; Bradley and Lang, 1994) to judge whether the presented emotion is more positive or more negative (valence). Second, they were asked to use SAM to judge to what degree the presented emotion triggers a reaction in them, in other words, to report their arousal level. Finally, the emotion attributions of participants were recorded through a multiple-choice task, where participants had to choose 1 label out of 11 to name the emotion of the stimuli they observed.

All participants answered the self-report Toronto Alexithymia Scale (TAS-20). This questionnaire measures 20 items with a five-point Likert scale, with a focus on identifying feelings, describing feelings, and externally oriented thinking (Bagby et al., 1994a,b). The two groups differed in terms of total TAS scores (*t* = 6.902, *p* < 0.001, *d* = 1.864), with higher alexithymia in iBPD (M = 69.00, SD = 8.94, SE = 1.660) than in controls (M = 52.89, SD = 8.31, SE = 1.63), with 24 and 4, respectively, being classified as alexithymic given the following interpretation: ≤51, no alexithymia; 52–60, borderline alexithymia; and ≥61, alexithymia. Levels of anxiety were measured in both populations using the State-Trait Anxiety Inventory (STAI; Spielberger, 2010). STAI state was higher for iBPD (M = 55.66, SD = 10.955, SE = 2.034) than for controls (M = 34.52, SD = 9.323, SE = 1.865) and so was the STAI trait (M = 58.72, SD = 10.42, SE = 1.94 and M = 41.76, SD = 9.02, SE = 1.80, respectively).

## Results

### Label Attribution

To investigate how facial displays were perceived in terms of emotion label attributions, unbiased hit rates, confusion matrices, and factorial analyses were computed. The “unbiased hit rate” (H_*u*_) was calculated as proposed by Wagner (1993) to account for response biases. H_*u*_ was calculated as the squared frequency of correct attributions for an emotion stimulus category divided by the product of the number of times the category was assessed and the overall frequency of this emotion label being attributed. Its value ranges from zero to one, one indicating that all stimuli of an emotion have been correctly identified and the respective emotion has never been falsely chosen for a different emotion.

**FIGURE 2:**
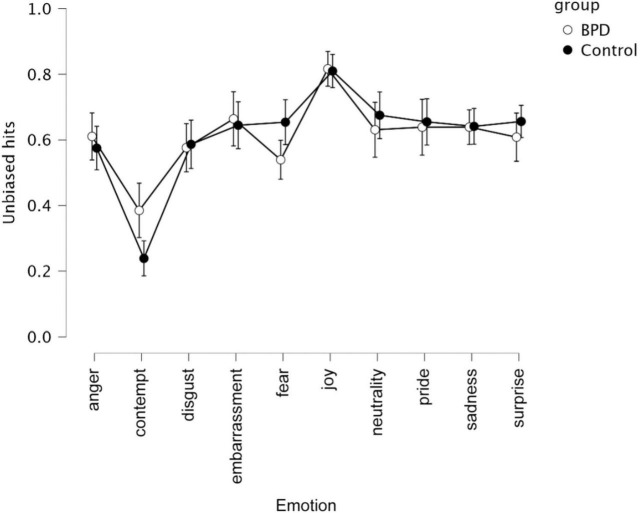
Unbiased hits for 2 groups (i.e., BPD, controls) and 10 emotions (i.e., anger, contempt, disgust, embarrassment, fear, joy, neutrality, pride, sadness, surprise).

**FIGURE 3:**
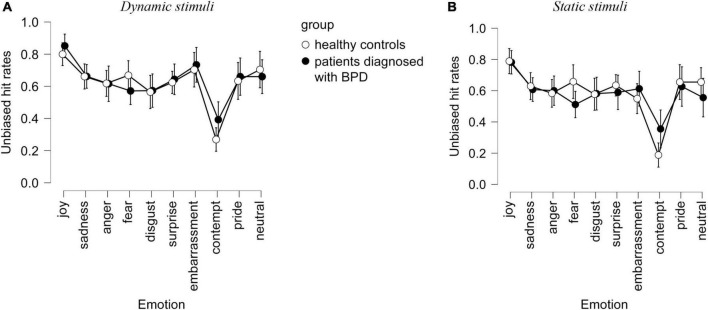
Unbiased hit rates observed in the interpretation of dynamic **(A)** and static **(B)** stimuli by healthy controls and in patients with BPD in terms of emotional labels. Confidence interval 95%.

A repeated-measures ANOVA on unbiased hit rates and STAI trait, STAI state, and TAS as covariates showed a participant group and emotion interaction effect, as well as an emotion effect (see Table 1 and Figure 2). There was also a group effect. No effects of the modality of stimuli were observed (Figure 3).

**TABLE 1:** Repeated measures ANOVA on the number of unbiased hits.

Cases	Sum of squares	df	Mean square	F	*p*	η^2^
**Within subjects effects**						
Modality	0.035	1	0.035	0.515	0.476	4.309e-4
Modality × group	0.031	1	0.031	0.461	0.500	3.854e-4
Modality × fear_state	0.014	1	0.014	0.210	0.649	1.758e-4
Modality × fear_trait	0.220	1	0.220	3.248	0.077	0.003
Modality × TAS_total	0.169	1	0.169	2.496	0.120	0.002
Residuals	3.449	51	0.068			
Emotion	1.582	9	0.176	2.241	0.019	0.020
Emotion × group	1.530	9	0.170	2.167	0.023	0.019
Emotion × fear_state	0.829	9	0.092	1.174	0.309	0.010
Emotion × fear_trait	0.324	9	0.036	0.459	0.902	0.004
Emotion × TAS_total	0.663	9	0.074	0.939	0.491	0.008
Residuals	36.008	459	0.078			
Modality × Emotion	0.201	9	0.022	0.511	0.867	0.002
Modality × Emotion × group	0.194	9	0.022	0.492	0.880	0.002
Modality × Emotion × fear_state	0.218	9	0.024	0.554	0.834	0.003
Modality × Emotion × fear_trait	0.093	9	0.010	0.236	0.989	0.001
Modality × Emotion × TAS	0.308	9	0.034	0.781	0.634	0.004
Residuals	20.072	459	0.044			
**Between subjects effects**						
Group	1.155	1	1.155	4.859	0.032	0.014
Fear_state	0.019	1	0.019	0.078	0.781	2.294e-4
Fear_trait	0.079	1	0.079	0.331	0.567	9.747e-4
TAS_total	1.498	1	1.498	6.302	0.015	0.019
Residuals	12.126	51	0.238			

*Type III sum of squares.*

**FIGURE 4:**
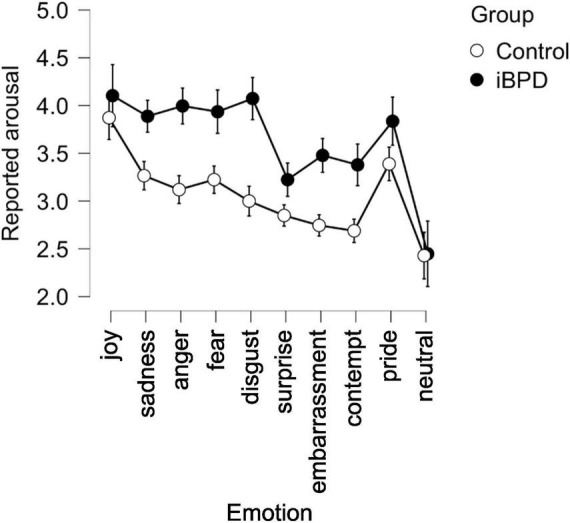
Arousal judgments in the control and iBPD population. Confidence interval 95%.

Contempt expressions were labeled as contempt more often by iBPD (H_*u*_ = 0.34 *M* = 2.11; SD = 1.26) vs. controls (Hu = 0.18, *M* = 2.11; SD = 1.26, *p* < 0.05). The misattribution profiles were slightly different (see Table 2), with the attribution of a surprise to the contempt expression significantly higher in controls (*p* < 0.05). Thus, controls misattributed the expression mostly to surprise (25%) and to the “none of the above” emotion category (22%). iBPD attributed the “none of the above” category most often (20%) than surprise (19%).

**TABLE 2:** Confusion matrix for **(A)** inpatients with BPD and **(B)** the control group.

		Label attributions										
		Anger	Contempt	Disgust	Embarrassment	Fear	Joy	Neutral	Pride	Sadness	Surprise	None
**(A) Confusion matrix for iBPD**												
Stimuli	Anger	75	5	4	1	1	0	1	0	7	2	5
	Contempt	1	44	3	5	0	0	4	0	4	19	19
	Disgust	18	10	67	0	0	0	0	0	1	2	2
	Embarrassment	0	3	1	73	1	0	3	1	9	3	6
	Fear	1	0	7	3	69	0	1	0	2	13	3
	Joy	0	0	0	0	0	93	2	1	0	0	2
	Neutral	2	5	1	1	1	0	73	0	9	0	6
	Pride	0	8	0	1	0	18	2	67	0	0	4
	Sadness	2	2	4	2	2	0	1	0	81	2	4
	Surprise	0	0	0	1	9	0	0	0	0	86	2
	Total	100	79	87	87	84	112	87	71	113	127	52
**(B) Confusion matrix for the control group**												
**Controls**												
Stimuli	Anger	75	5	3	1	0	0	1	0	10	2	2
	Contempt	2	35	2	7	0	0	3	0	4	24	22
	Disgust	20	8	67	0	0	0	0	0	0	2	2
	Embarrassment	0	1	1	73	0	0	3	1	9	6	7
	Fear	2	0	5	3	69	0	0	0	3	15	2
	Joy	0	0	0	1	0	94	4	1	0	0	0
	Neutral	2	4	0	1	0	0	78	0	8	0	5
	Pride	0	6	0	1	0	19	2	69	0	0	2
	Sadness	1	3	3	2	0	0	0	0	83	2	3
	Surprise	2	0	0	1	1	0	0	0	1	93	0
	Total	104	63	82	90	73	114	92	73	118	147	44

*Rounded percentages of label responses (%) attributed to each emotion stimulus category.*

The overattribution of surprise labels in controls went to contempt and fear: 25% of all contempt stimuli were labeled as surprise as well as 16% of all fear stimuli.

In iBPD and controls, anger stimuli were most often mislabeled as sadness (7%; 9%), disgust as anger (18%; 20%), and contempt as surprise (19%; 25%).

In iBPD and controls, the anger label was most often misattributed to disgust (18%; 20%), disgust was most often misattributed to fear (8%; 5%), while contempt to disgust (9%; 9%), pride (8%; 6%), and anger (5%; 5%).

### Arousal

The repeated measures ANOVA (Table 3) showed differences between groups of participants in the arousal reports (Figure 4), with TAS, STAI trait, and STAI state as covariants. There was an emotion effect, an emotion × group interaction but no effect of modality nor any group × emotion × modality interaction.

**TABLE 3:** Repeated measures ANOVA on arousal levels and mean scores per category.

Cases	Sum of squares	df	Mean square	F	*p*	η^2^
**Arousal scores: repeated measures ANOVA**						
**Within subjects effects**						
Dynamics	0.529	1	0.529	2.838	0.098	2.352e-4
Dynamics × group	0.398	1	0.398	2.137	0.15	1.771e-4
Dynamics × fear_state	0.19	1	0.19	1.019	0.318	8.446e-5
Dynamics × fear_trait	0.9	1	0.9	4.827	0.033	4.001e-4
Dynamics × TAS_total	0.133	1	0.133	0.713	0.402	5.909e-5
Residuals	9.506	51	0.186			
Emotion	27.384	9^a^	3.043^a^	3.312^a^	<0.001^a^	0.012
Emotion × group	20.542	9^a^	2.282^a^	2.484^a^	0.009^a^	0.009
Emotion × fear_state	6.421	9^a^	0.713^a^	0.777^a^	0.638^a^	0.003
Emotion × fear_trait	13.645	9^a^	1.516^a^	1.65^a^	0.099^a^	0.006
Emotion × TAS_total	6.951	9^a^	0.772^a^	0.841^a^	0.579^a^	0.003
Residuals	421.69	459	0.919			
Dynamics × Emotion	1.189	9^a^	0.132^a^	0.581^a^	0.813^a^	5.287e^a^-4
Dynamics × Emotion × group	1.992	9^a^	0.221^a^	0.974^a^	0.461^a^	8.857e-4
Dynamics × Emotion × fear_state	0.838	9^a^	0.093^a^	0.41^a^	0.93^a^	3.729e-4
Dynamics × Emotion × fear_trait	0.858	9^a^	0.095^a^	0.419^a^	0.925^a^	3.814e-4
Dynamics × Emotion × TAS_total	0.577	9^a^	0.064^a^	0.282^a^	0.979^a^	2.567e-4
Residuals	104.293	459	0.227			
**Between subjects effects**						
**Cases**						
Group	82.398	1	82.398	2.748	0.104	0.037
Fear_state	1.862	1	1.862	0.062	0.804	8.281e-4
Fear_trait	16.560	1	16.560	0.552	0.461	0.007
TAS_total	0.736	1	0.736	0.025	0.876	3.271e-4
Residuals	1529.136	51	29.983			

**Dynamics**	**Emotion**	**Group**	**Mean**	**SD**	***N***

**Descriptives**					
Dynamic	Neutral	BPD	2.214	1.265	28
		Control	2.429	1.073	28
	Pride	BPD	3.938	1.182	28
		Control	3.512	1.410	28
	Sadness	BPD	3.929	1.359	28
		Control	3.616	1.569	28
	Surprise	BPD	3.196	1.377	28
		Control	2.938	1.241	28
	Anger	BPD	4.045	1.634	28
		Control	3.159	1.393	28
	Contempt	BPD	3.446	1.436	28
		Control	2.759	1.250	28
	Disgust	BPD	4.071	1.611	28
		Control	3.018	1.422	28
	Embarrassment	BPD	3.420	1.328	28
		Control	2.884	1.148	28
	Fear	BPD	3.902	1.425	28
		Control	3.321	1.409	28
	Joy	BPD	4.170	1.421	28
		Control	3.985	1.718	28
Static	Neutral	BPD	2.625	1.449	28
		Control	2.496	1.088	28
	Pride	BPD	3.598	1.286	28
		Control	3.375	1.389	28
	Sadness	BPD	3.938	1.353	28
		Control	3.438	1.430	28
	Surprise	BPD	3.188	1.431	28
		Control	2.982	1.333	28
	Anger	BPD	3.813	1.595	28
		Control	3.202	1.398	28
	Contempt	BPD	3.268	1.350	28
		Control	2.820	1.181	28
	Disgust	BPD	4.089	1.645	28
		Control	3.268	1.450	28
	Embarrassment	BPD	3.420	1.198	28
		Control	2.759	1.066	28
	Fear	BPD	3.929	1.672	28
		Control	3.286	1.422	28
	Joy	BPD	3.946	1.628	28
		Control	3.857	1.836	28

*Type III sum of squares.*

*^a^Mauchly’s test of sphericity indicates that the assumption of sphericity is violated (p < 0.05).*

*Type III sum of squares.*

Both populations reported the strongest arousal for joy (M = 4.05; SD = 1.67). In iBPD, this was followed by high arousal scores for disgust (M = 4.0; SD = 1.59), followed closely by anger (M = 3.90; SD = 1.9). In controls, arousal levels that followed those of joy were for pride (M = 3.71; SD = 1.75) and sadness (M = 3.29; SD = 1.56).

### Valence

To check the differences between groups of participants in the emotional valence attribution, repeated-measures ANOVA was computed, using TAS, STAI trait, and STAI state as covariants.

**FIGURE 5:**
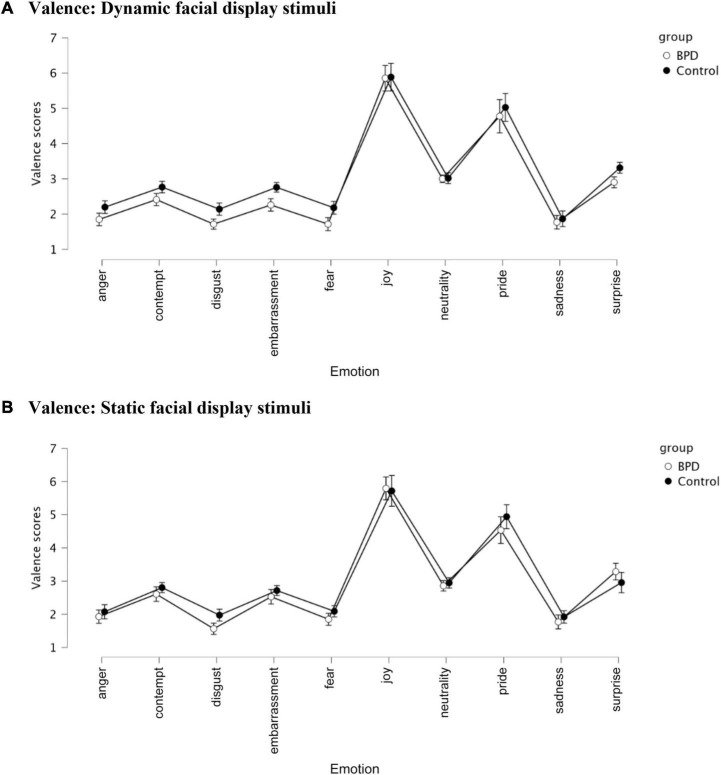
Valence judgments in the control and iBPD population and 10 emotions (i.e., anger, contempt, disgust, embarrassment, fear, joy, neutrality, pride, sadness, and surprise). **(A)** Valence: Dynamic facial display stimuli. **(B)** Valence: Static facial display stimuli.

There was an emotion effect (*p* < 0.001) as well as a modality × emotion × group interaction (*p* = 0.002) (see Table 4 and Figure 5). The modality × group interaction did not reach significance (*p* = 0.051).

**TABLE 4:** Repeated measures ANOVA on valence scores and mean scores per category.

Cases	Sum of squares	df	Mean square	F	*p*	η^2^
**Valence scores: repeated measures ANOVA**						
**Within subjects effects**						
Dynamics	0.002	1	0.002	0.016	0.901	4.564e-6
Dynamics × group	0.631	1	0.631	4.009	0.051	0.001
Dynamics × fear_state	0.093	1	0.093	0.592	0.445	1.738e-4
Dynamics × fear_trait	0.199	1	0.199	1.267	0.266	3.720e-4
Dynamics × TAS_total	0.026	1	0.026	0.164	0.687	4.813e-5
Residuals	8.024	51	0.157			
Emotion	41.832^a^	9^a^	4.648^a^	6.715^a^	<0.001 ^a^	0.078
Emotion × group	6.51^a^	9^a^	0.723^a^	1.045^a^	0.403^a^	0.012
Emotion × fear_state	1.729^a^	9^a^	0.192^a^	0.277^a^	0.981^a^	0.003
Emotion × fear_trait	2.21^a^	9^a^	0.246^a^	0.355^a^	0.956^a^	0.004
Emotion × TAS_total	2.379^a^	9^a^	0.264^a^	0.382^a^	0.944^a^	0.004
Residuals	317.695	459	0.692			
Dynamics × Emotion	1.366^a^	9^a^	0.152^a^	1.017^a^	0.425^a^	0.003
Dynamics × Emotion × group	2.988^a^	9^a^	0.332^a^	2.225^a^	0.02^a^	0.006
Dynamics × Emotion × fear_state	1.412^a^	9^a^	0.157^a^	1.052^a^	0.398^a^	0.003
Dynamics × Emotion × fear_trait	1.418^a^	9^a^	0.158^a^	1.056^a^	0.394^a^	0.003
Dynamics × Emotion × TAS_total	1.097^a^	9^a^	0.122^a^	0.817^a^	0.601^a^	0.002
Residuals	68.476	459	0.149			
**Between subjects effects**						
**Cases**						
Group	0.412	1	0.412	0.280	0.599	7.686e-4
Fear_state	2.126	1	2.126	1.444	0.235	0.004
Fear_trait	0.071	1	0.071	0.048	0.827	1.329e-4
TAS_total	0.341	1	0.341	0.232	0.632	6.365e-4
Residuals	75.060	51	1.472			

**Dynamics**	**Emotion**	**Group**	**Mean**	**SD**	***N***

**Descriptives**					
Dynamic	Neutral	BPD	3.000	0.425	28
		Control	3.018	0.425	28
	Pride	BPD	4.777	1.301	28
		Control	5.027	1.019	28
	Sadness	BPD	1.768	0.466	28
		Control	1.866	0.567	28
	Surprise	BPD	2.902	0.483	28
		Control	3.313	0.460	28
	Anger	BPD	1.848	0.562	28
		Control	2.196	0.524	28
	Contempt	BPD	2.411	0.562	28
		Control	2.766	0.479	28
	Disgust	BPD	1.714	0.439	28
		Control	2.140	0.568	28
	Embarrassment	BPD	2.259	0.579	28
		Control	2.759	0.483	28
	Fear	BPD	1.714	0.517	28
		Control	2.179	0.531	28
	Joy	BPD	5.857	0.939	28
		Control	5.887	0.974	28
static	Neutral	BPD	2.857	0.520	28
		Control	2.946	0.399	28
	Pride	BPD	4.536	1.140	28
		Control	4.943	0.917	28
	Sadness	BPD	1.768	0.581	28
		Control	1.920	0.509	28
	Surprise	BPD	3.286	0.637	28
		Control	2.955	0.788	28
	Anger	BPD	1.929	0.600	28
		Control	2.074	0.533	28
	Contempt	BPD	2.607	0.672	28
		Control	2.804	0.483	28
	Disgust	BPD	1.563	0.470	28
		Control	1.973	0.542	28
	Embarrassment	BPD	2.527	0.629	28
		Control	2.714	0.535	28
	Fear	BPD	1.848	0.492	28
		Control	2.089	0.487	28
	Joy	BPD	5.795	0.855	28
		Control	5.720	1.159	28

*Type III sum of squares.*

*^a^Mauchly’s test of sphericity indicates that the assumption of sphericity is violated (p < 0.05).*

*Type III sum of squares.*

## Discussion

When studying BPD, researchers have focused until now on the recognition of emotions, i.e., the attribution of an expected emotional label to a specific expression, and sensitivity to threat, i.e., interpretation of facial displays of anger or sometimes fear. The aim of this study was to change the focus to moral emotions (Hutcherson and Gross, 2011). These emotions, namely, anger, contempt, and disgust, are particularly relevant to iBPD given that a strong fear of rejection is a diagnostic feature of BPD (Goodman et al., 2014). In line with former studies describing comorbidities associated with BPD, we recorded and anxiety scores in all participants as their levels are known to influence emotion interpretation and define perception biases.

As expected, we observed a more accurate interpretation of contempt in iBPD compared with controls. These results could be explained by these emotional displays being perceived by individuals as predictors of rejection and abandonment, therefore, having survival value for this population and necessitating correct interpretation. The high sensitivity to the displays of contempt may also be related to their vulnerability to harm from others as well as their generalized belief that other individuals are hostile (Beck and Freeman, 1990).

As expected, dynamic presentations improved recognition of emotions in both populations, especially for the emotion of contempt, which is a complex emotion often poorly recognized (see former uses of the ADFES database in the general population: Van der Schalk et al., 2011; Wingenbach et al., 2016).

To summarize, our results support that iBPD have good emotion recognition skills, on par with healthy individuals, except facial expressions of contempt, which are recognized more accurately by iBPD, most probably due to their survival value iBPD attributes to this social information.

Confirming former studies reporting sensitivity to threat, iBPD, compared with healthy controls, attributed more negative valence to all presented emotions, more specifically, contempt, embarrassment, fear, disgust, and surprise.

The iBPD reported higher arousal to observed stimuli than healthy individuals, which means a higher reactivity to facial expressions. The highest arousal was observed in dynamically presented emotions of anger and disgust. This is in line with numerous studies showing that iBPD tend to react more strongly to anger expressions and to judge strangers performing simple tasks such as entering a room and sitting down as more aggressive than healthy controls do (Barnow et al., 2009). This bias might be explained by the belief expressed by iBPD that all other individuals are malevolent (Pretzer, 1990; Arntz and Veen, 2001).

Greater emotional reactivity seen in BDP controls has also been observed in their brain activity during the perception of negative social signals (van Zutphen et al., 2015), particularly greater activation of the amygdala during the perception of facial expressions of anger (Donegan et al., 2003; Minzenberg et al., 2007). The high arousal induced by disgust expressions in iBPD has previously been reported (Bland et al., 2004; Guitart-Masip et al., 2009; Jovev et al., 2011), and according to Rusch et al. (2011), not only the perceived disgust but also the experience of disgust toward the self may be a prominent emotion in BPD pathology, stronger than anxiety or anger.

The general greater emotional sensitivity reflected in the high arousal levels reported by iBPD could be an expression of non-adaptive emotion regulation strategies, such as the less frequent redirection of attention from negative to more positive stimuli (Porter et al., 2016).

BPD is an important psychological disorder characterized by emotional, interpersonal, and behavioral instability (American Psychiatric Association [APA], 2013). Following the biosocial theory proposed by Linehan (1993), iBPD can exhibit difficulties with the identification and correct reaction to relevant social stimuli, particularly to the facial expressions of emotions of others. This might be one of the factors contributing to difficulties in interpersonal functioning.

Our results support previous studies suggesting a higher sensitivity to negative emotions (Lynch et al., 2006; Zanarini and Frankenburg, 2007) and are in line with reports of iBPD exhibiting higher emotional reactivity (Ebner-Priemer et al., 2007). This higher mood lability and emotional fluctuations in iBPD could explain some of the previously reported divergent results reported in different studies, e.g., neutral faces being colored by own emotional states of patients (e.g., Wagner and Linehan, 1999; Herr and Meier, 2020). In this study, we observed no higher attribution of negative valence to neutral faces, which is aligned with findings from several studies failing to find any significant differences in neutral facial expression recognition accuracy (Levine et al., 1997; Bland et al., 2004; Minzenberg et al., 2006a,b; Merkl et al., 2010; Mier et al., 2012; Hagenhoff et al., 2013).

Given how important it is to use due to their better ecological validity and the added complexity seen in a naturalistic emotional context (e.g., Dziobek, 2012; Hyniewska et al., 2019) and the fact that previous studies on emotion perception in iBPD mostly relied on static stimuli, we introduced dynamic stimuli, and their comparison with static ones, to try to elucidate discrepancies observed in iBPD and emotion labeling of facial expressions. Our study did not show any particular advantage of modality for iBPD; however, more studies are necessary to understand the process at play and whether any conditions (e.g., for low-intensity emotions) require iBPD to rely on dynamic vs. static information in facial emotion interpretation tasks. Intensity of facial expressions is a factor that will need to be integrated in future studies, especially for complex emotions such as contempt (see Wingenbach et al., 2016).

To comprehend the conflicting results regarding emotion interpretation in iBPD, the great heterogeneity of this clinical population needs to be acknowledged (Mitchell et al., 2014). Numerous factors could play a role in influencing the performance of patients, from the emotional and clinical state of the individual to comorbidities. Mitchell et al. (2014) suggested that emotional states and personal experience could be influencing emotion interpretation in iBPD. For example, following the mood-congruency hypothesis (Bower, 1981), patients with BPD who regularly experience negative states might be more skilled at processing and interpretating negative stimuli.

Given data on neglect and childhood abuse often reported in iBPD (Wagner and Linehan, 1999), which are considered factors influencing the shaping of the borderline traits, further studies on emotion interpretation would need to record these characteristics for this population and healthy counterparts. This is on par with the quantifying of the degree of BPD-related dysfunctions, along with the study of BPD traits in non-clinical populations (Trull et al., 1997).

The sensitivity to negativity and more generally emotional reactivity observed in patients with BPD is in line with Linehan’s biosocial model of emotion dysregulation. This dysregulation can be explained by an interplay of biological vulnerabilities and an early environment characterized by invalidation (Linehan, 1993). Particularly, sensitivity to injustice predisposes to emotional and cognitive biases and to intense reactions when expecting and perceiving potential rejection (Downey and Feldman, 1996) either as a victim, an observer, or a perpetrator (Schmitt et al., 2010). Furthermore, sensitivity to moral disgust predisposes to a stronger experience of disgust when confronted with moral norm violations (Tybur et al., 2009). These sensitivities could help explain cognitive and emotional biases observed in individuals with high BPD scores, who show a tendency to ascribe negative and hostile intent to observed social interactions and more generally ambiguous or explicit behavior of others (De Panfilis et al., 2015). Possibly, the development of atypical coping strategies, including emotional perception biases, might be functional attempts to deal with the fear of abandonment and emotional overstimulation, which in specific life circumstances might appear to be effective coping.

## Data Availability Statement

The original contributions presented in the study are included in the article/Supplementary Material, further inquiries can be directed to the corresponding authors.

## Ethics Statement

The studies involving human participants were reviewed and approved by Department of Biological and Behavioral Psychology, Behavioral Neuroscience Lab, SWPS University of Social Sciences and Humanities, Warsaw, Poland. The patients/participants provided their written informed consent to participate in this study. The animal study was reviewed and approved by Department of Biological and Behavioral Psychology, Behavioral Neuroscience Lab, SWPS University of Social Sciences and Humanities, Warsaw, Poland.

## Author Contributions

SH and KR were responsible for the conceptual definition of the research. JD obtained the data. SH, JD, and KR analyzed the data. All authors wrote the manuscript.

## Conflict of Interest

The authors declare that the research was conducted in the absence of any commercial or financial relationships that could be construed as a potential conflict of interest.

## Publisher’s Note

All claims expressed in this article are solely those of the authors and do not necessarily represent those of their affiliated organizations, or those of the publisher, the editors and the reviewers. Any product that may be evaluated in this article, or claim that may be made by its manufacturer, is not guaranteed or endorsed by the publisher.
